# Selected In Situ Hybridization Methods: Principles and Application

**DOI:** 10.3390/molecules26133874

**Published:** 2021-06-24

**Authors:** Dominika Veselinyová, Jana Mašlanková, Katarina Kalinová, Helena Mičková, Mária Mareková, Miroslava Rabajdová

**Affiliations:** 1 Department of Medical and Clinical Biochemistry, Faculty of Medicine, Pavol Jozef Šafárik University in Košice, Trieda SNP 1, 04011 Košice, Slovakia; veselinyova.dominika@gmail.com (D.V.); katarina.kalinova@student.upjs.sk (K.K.); maria.marekova@upjs.sk (M.M.); miroslava.rabajdova@upjs.sk (M.R.); 2 Department of Medical Biology, Faculty of Medicine, Pavol Jozef Šafárik University in Košice, Trieda SNP 1, 04011 Košice, Slovakia; helena.mickova@upjs.sk

**Keywords:** fluorescent in situ hybridization, DNA/RNA imaging, application of FISH

## Abstract

We are experiencing rapid progress in all types of imaging techniques used in the detection of various numbers and types of mutation. In situ hybridization (ISH) is the primary technique for the discovery of mutation agents, which are presented in a variety of cells. The ability of DNA to complementary bind is one of the main principles in every method used in ISH. From the first use of in situ techniques, scientists paid attention to the improvement of the probe design and detection, to enhance the fluorescent signal intensity and inhibition of cross-hybrid presence. This article discusses the individual types and modifications, and is focused on explaining the principles and limitations of ISH division on different types of probes. The article describes a design of probes for individual types of in situ hybridization (ISH), as well as the gradual combination of several laboratory procedures to achieve the highest possible sensitivity and to prevent undesirable events accompanying hybridization. The article also informs about applications of the methodology, in practice and in research, to detect cell to cell communication and principles of gene silencing, process of oncogenesis, and many other unknown processes taking place in organisms at the DNA/RNA level.

## 1. Introduction

Hybridization of nucleic acids is one of the most widely used tools in molecular biology and therefore is applied in several techniques in diagnostics and research. This technique recognizes DNA and RNA targets which can be visualized with two types of probes, fluorescent (fluorescent in situ hybridization; FISH) or chromogen (chromogenic in situ hybridization; CISH), based on the same procedure principle. The subtypes of ISH are so wide that it is necessary to select some the most important ISH methods recommended to use as a specific diagnostic tool.

The principle of ISH is based on the complementary function of DNA/RNA to bond each of their basis pair [1,2]. The procedure of ISH determination involves several steps: the first step is preparation of a specific probe (the majority or procedures use commercial ISH probes in routine diagnostics), then application of probe on a sample, native or fixed, follows. In the third step, a sample is denatured, further renatured, and finally hybridized with probe overnight [3,4]. During the hybridization process, a shorter strand of nucleic acid is defined as a probe, which complementary binds to target sequences of the examined gene. The last step is detection of the probe itself using a specific software or a fluorescence imaging microscope [5,6]. 

The separation of complementary DNA strands can be achieved by: (1) the action of temperature (30–100 °C depending on the content of GC-pairs which increase the temperature required to denature DNA); (2) a change in concentration of salts in solution, the reduction of salt removes ions that provide “compensation” of negative charges in the DNA molecule which leads to the point where DNA can be denatured at lower temperature; and (3) extreme pH values and the presence of organic compounds, like dimethyl sulfoxide and formamide, which interrupts hydrogen bonds between DNA strands. The length of target sequence and presence of unpaired bases are factors that affect the denaturation time [3,5,7,8]. A subsequent restoration of normal conditions allows renaturation of nucleic acid [6,9]. 

The denaturation of DNA is accompanied by a hyperchromic effect; the UV absorbance of DNA absorbs UV light at 260 nm and increases with the cleavage of DNA from double-stranded to single-stranded. This hyperchromicity has been used to increase the sensitivity of DNA visualization, and changes in the base arrangement during denaturation increases light absorption [9,10]. This phenomenon is caused by the breakdown of double-stranded DNA molecules into single-stranded molecules, the latter of which absorb UV radiation more than double-stranded molecules [9,10]. After denaturation, the probe is incorporated into target DNA/RNA, then renatured and hybridized overnight. The next day, the procedure continues with the post-hybridization rinse which erases a non-specific binding of cross-hybrids in the samples and minimizes the false background signals [5,7]. The purpose of this review is to draw and characterize common ISH variants and their advantages together to be useful in the diagnostic determination of suitable embryos in preimplantation diagnostics, or in the field of prenatal and postnatal genetic diagnostics in humans.

## 2. Probes Used in In Situ Hybridization

The challenge in developing various ISH methods is to improve the sensitivity of the probes used to visualize the target sequence. Probes are visualized: (1) via radioisotope labelling methodology, which is considered to be very sensitive, but has a short half-life, long exposure, and health risk [8,11]; (2) via non-radiolabelled probes, and the use of poly(rA)-/poly(dT)-antibodies for analysis of RNA-DNA hybrids in chromosomes (releasing a signal by rhodamine-conjugated secondary antibody, the other option is rRNA-biotin approach that uses indirect detection by using avidin based detection); (3) by using biotin to mark DNA (using nick translation, which was detected directly by fluorescence or indirectly by enzymatically conjugated secondary antibodies); (4) by probes labelled during the synthesis of DNA (allowed to bind a sufficient amount of fluorophore to the target DNA sequence and creates direct labelling); and (5) via digoxigenin labelling by classic PCR and degeneration of oligonucleotides [6,12]. Fluorescent probes used in interphase FISH are able to detect genetic aberration in range of 150~200 kb, but are unable to detect smaller micro-deletions presented in the genome. Therefore, scientists are focused on the creation of methods to improve detection limits [8,13,14,15].

The great advantage of the ISH procedures is the ability to visualize a target sequence in all cell types and in various types of tissue [8]. The four primary reasons that recommend hybridization over other methods used in diagnostic and scientific research are the simplicity of processing, specificity of the monitored nucleic sequence, simple interpretation of the obtained results, and the unlimited application to interphase cells and metaphases, as well as paraffin and formalin fixed tissue sections. The in situ hybridization probes are distinguished by different factors, for example by size of the fragments, by nucleic acid type (DNA/RNA), and localization of the probe on the chromosomes [2,3,8].

The most important part of ISH is the design of right kind of a probe. A probe is defined by the specific sequence of nucleic acid that is in single-stranded DNA/RNA, with characteristic features such as its affinity to the target sequence of nucleic acid [8,12]. The probe and target sequence bind to each other based on the principle of complementarity. The probes are divided in terms of their composition and nature into: (1) homogeneous—the probe is made by one sequence; (2) heterogeneous—consisting of mixtures of two or more sequences [6]; or (3) by the type of nucleic acid into DNA or RNA. Another way to divide probes is by their method of incorporation into the target nucleic acid sequence: (1) radiolabelling; (2) hapten incorporation; and (3) direct enzyme labelling [6,12]. The probes are also divided according to their stability, into isotopic and non-isotopic probes [6,8,16]. The classification of probes, depending on labelling distribution, divide probes in several groups (Table 1).

**Figure 1:**
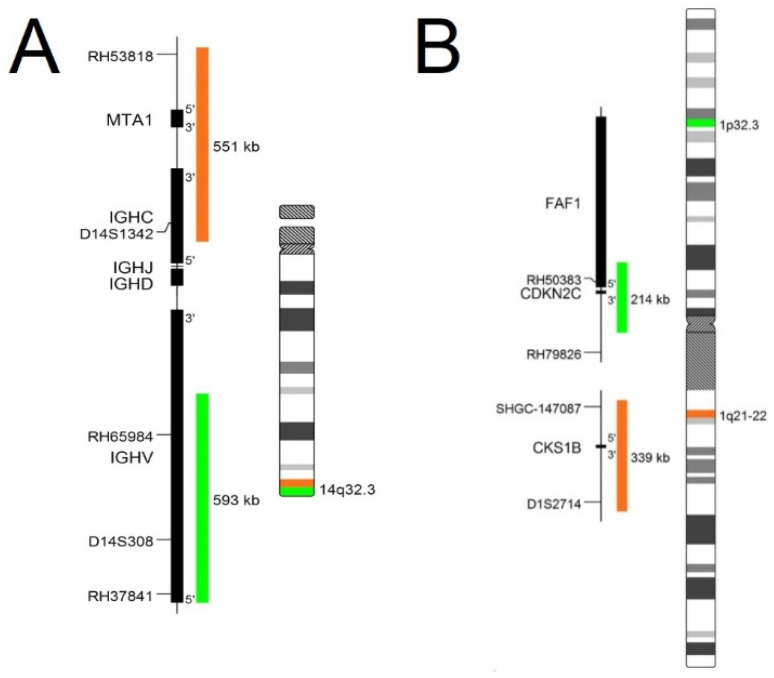
Probe map of chromosome 14 and chromosome 1 with visualization of target genes, their sizes, and fluorescent dye used on visualization. (**A**) Map of break-apart probe of IGH gene capable of detected aberration: deletion, amplification, and breakage in gene; (**B**) Map of numerical probe on chromosome 1 able to detect only numerical changes: deletion and additions of material [17].

In routine diagnostics, the commercial probes are supplied with an information list such as the chromosome map illustrated in Figure 1. The map visualizes the exact localization of probes, genes that are examined, and the type of fluorescent dyes used in probe design. The information list also contains recommended FISH protocol (temperature protocol, necessary equipment, and a list of chemicals). The information sheet shows the expected results of schemes with normal and aberrant interphase nuclei of detected cells. Figure 1 shows two types of probes: (A) is a break-apart probe which informs about the localization of break points in a gene, and (B) is a numerical probe which informs about numerical change such as additions or deletions of genes which detect quantitative changes in fluorescent signals (partial deletion, or in opposition, partial enhancement of the signal). Deletion or amplification is occasionally not shown in a whole visualized area by fluorescent dye, however this only occurs in specific parts of genes [17]. 

## 3. Types of In Situ Hybridization

Around the world, diagnostic laboratories are using ISH as an efficient and fast tool to identify chromosomal aberrations and/or changes on a genetic level such as the addition and deletion of specific genes that play a significant role in diagnostics [2,18,19]. FISH is a golden standard in the field of diagnostics of oncological diseases due to the creation of typical FISH panels for individual types of diseases. These panels contain the most common genetic changes that have been observed in individual types of diseases [2,20]. The possibility of applying FISH to both native and fixed slides will allow the identification of genetic changes and chromosomal aberrations in various types of tissue and cells. Therefore, this diagnosis is an ideal choice in terms of resolution, detection, and evaluation speed of diagnostic procedure [18,21].

### 3.1. DNA In Situ Hybridization of the Specific Genes

DNA in situ hybridization is used to identify the position of genes and localize and detect the specific DNA sequences in cells. There are many types of ISH laboratory techniques in which a single-stranded DNA probe is complementary paired with target gene. In the section below, we describe several of the most prominent methods of interphase FISH or Multiplex ISH.

#### 3.1.1. Interphases Fluorescent In Situ Hybridization 

**Figure 2:**
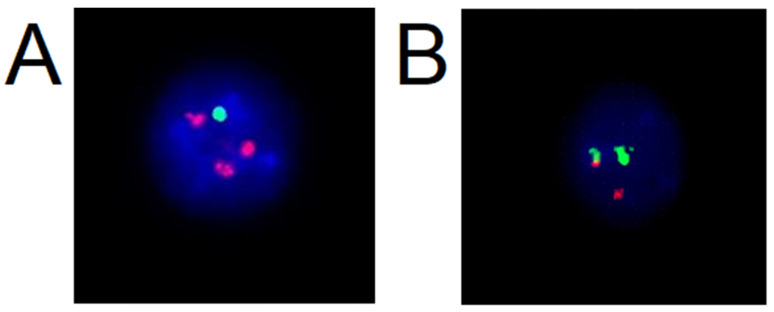
Interphase nuclei with fluorescence numerical probe CDKN2C/CKS1B and break apart probe on 14q32 imaging IGH- Immunoglobulin Heavy Locus Gene: (**A**) aberrant nuclei with deletion in CDKN2C and gains of CKS1B gene, (**B**) aberrant nuclei with chromosome break in IGH locus (source: Author with permission of Medirex laboratory).

Interphase FISH is type of fluorescent in situ hybridization technique which is applied to interphase nuclei; it is not necessary to cultivate a sample for cell division and form a metaphase spread [2]. This type of FISH is widely used in routine oncology diagnostics. In haemato-oncological diagnostics, the FISH method is used on peripheral blood and bone marrow samples; biological material is cultivated (stimulated), or unstimulated with grow factors, then processed by triple fixation using a mixture of glacial acetic acid and methanol [16,20]. The sample is fixed on a slide to which is applied a commercially available probe with the target sequence to be monitored and identified. Figure 2 shows specific findings presented in interphase nuclei of classic FISH with the use of a commercially available probe. Figure 2 displays nuclei with amplification of examined gene (Figure 2A) and within break in gene (Figure 2B). When a breakage is detected in a gene, the procedure continues in search of a translocation partner for the gene with respect to the most common presence of translocation in the specific disease [16]. 

**Figure 3:**
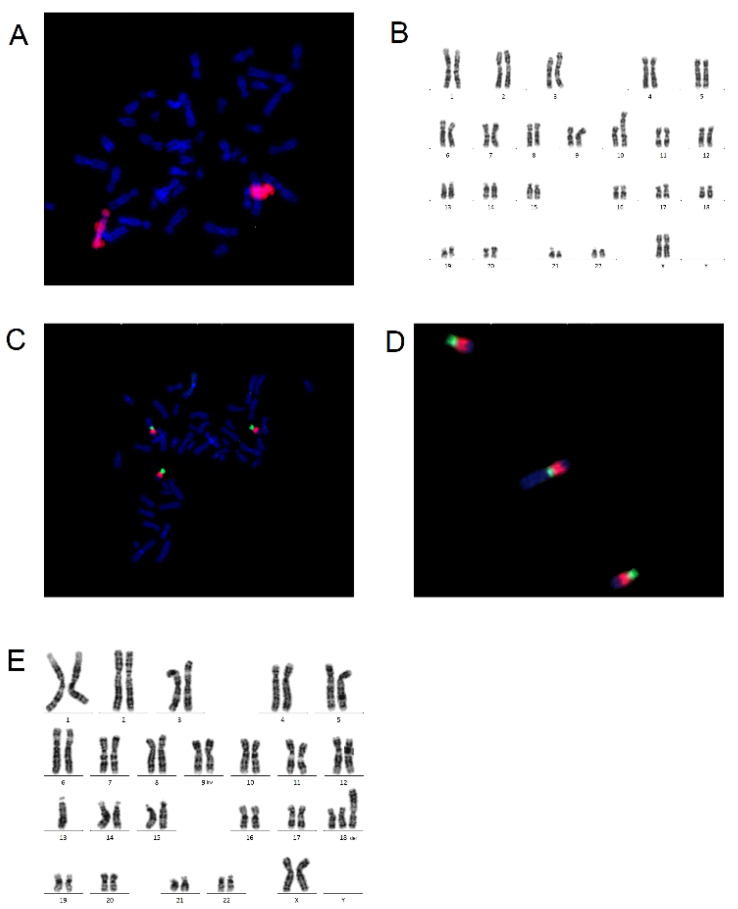
Metaspread with use of whole chromosome painting-WCP and M-band and karyotypes: (**A**) metaspread with WCP10 with orange dye shows gain of genetic information from chromosome 10; (**B**) karyotype on patients with duplication of long arm of chromosome 10: 46, XX, dup (10) (q11q26); (**C**) multicolor chromosome banding probe on chromosome 18; (**D**) zoomed picture of 3 chromosomes 18 with M-band probes; and (**E**) karyotype of newborn with trisomy of 18 presented in rare form: 46, XX, der (13;18) (q10; p10) +18 (source: Author with permission of Medirex laboratory).

In the diagnostic of balanced translocation modification of this type of FISH is used. A probe is applied to a metaphase spread to identify the location of genes on chromosomes [20], or a probe which dyes a whole chromosome is utilized to visualize all genes on chromosome, or an M-band probe is used for the identification of translocation on one chromosome and for the detection of different types of rearrangements detected on only one chromosome [20,22]. The principle of multicolor chromosome banding is based on a series of partial chromosome paints, identifying a different region of a single chromosome. An M-band probe colors every band on chromosome with different color which allows detection of intra chromosomal rearrangements [22]. In Figure 3, use of whole chromosome painting probes on a metaphase spread in a sample of amniotic fluid is shown. Figure 3A shows detected duplication of long arms of chromosome 10 visualized with orange-fluorescent dye: a patient with suspected genetic defect in their fetus. Figure 3B shows karyotype of the fetus: 46, XX, dup (10) (q11q26). Figure 3C,D present use of M-band multicolor chromosome banding on chromosome 18 in a new-born. Figure 3E displays the karyotype of the patient with duplication of long arms of chromosome 18 and translocation of chromosome 13 and 18.

The use of different colors creates different FISH techniques: Combinatorial Binary Ratio Labelling—COBRA FISH, which uses 24 different human chromosomes colored with five basic fluorophores. Due to the color combination of these five dyes, other color variants can be distinguished and also identify intra- and inter-chromosomal rearrangements [23,24]. 

Another technique is spectral karyotyping—SKY FISH, which allows a visualization of a whole genome by using a different color. Interpretation is proceeded by the classification of every single chromosome of a karyotype, and the comparison of all aberrations presented and summarized in the karyotype [16,25]. 

Multiplex FISH (M-FISH) uses the ability to identify 24 human chromosomes in a metaphase spread by hybridization labelled in different combinations of specific probes. The only difference between M-FISH and SKY is that M-FISH uses filter-based system to separate images sequentially from each other, and individual fluorochromes are then combined to generate the final image [14,26,27]. 

Primed in situ Labelling (PRINS) is a type of in situ hybridization presented with multiple advantages over the classical FISH. Chromosome identification is mediated due to the in situ binding of specific double stranded oligonucleotide primers, labelled forward and reverse into the target sequence in multiple cycles of amplification using multicolored labelling and enzymatic detection; therefore PRINS is a more specific and faster methodology than classic FISH diagnostics due to faster results compared to detection of every single gene on chromosome. Primed in situ Labelling is used for the detection of long repeating sequences of DNA satellites and telomeres, and in the detection of microdeletion syndromes presented in human embryos [16,28].

#### 3.1.2. Highly Multiplex In Situ Hybridization

In the era of next generation sequencing and arrays, comparative genomic hybridization FISH is considered a gold standard for the detection of the deletion and amplification of genes due to its ability to detect copy number quantification on a cellular level [29]. Scientists are focused on increasing multiplicity to rapidly determine copy numbers, which is highly necessary in cancer diagnostics due to the huge number of copies in genes observed in specific types of cancer [30]. The fact that FISH is capable of distinguishing minor subpopulations between cells, and therefore detect the mosaicism presented in these populations, makes FISH more applicable over robust methods such as new generation sequencing (NGS) and aCGH. These methods are limited and unable to determine these subpopulations [31,32]. As we mentioned above, the application of different combinations of fluorescence colors serves the detection of complex changes in the metaphase spread. The same principles of combinations of different colors are used in Highly Multiplex in situ Hybridization, albeit with some improvement, and with application of the latest knowledge in the fields of digital imaging and data processing. The limitation of previous multi-colored FISH methods was their application being limited to a metaphase spread, and the inability to detect changes in cells and tissues [30,31]. Highly Multiplex ISH consists of three steps: (1) probe construction and hybridization; (2) image acquisition; and (3) analysis of images. The creation of probes with target sequences of genes and combinations of different colors is followed by the nick translation incorporation of the probe and hybridization [31,32]. After hybridization, the hybridization protocol is followed by post hybridization washing and image preparation with a confocal microscope. For the detection of fluorescence color, lambda mode unmixing with a 34-channel photomultiplier tube for high-resolution spectral image acquisition is used [30,32], and results are stored in the spectra-database of the microscope. For image evaluation, it is necessary to use the algorithm with the entire emission spectra of each fluorescent color [32,33]. This algorithm helps to determine the number of gene copies in the nuclei from the optical section used in 3D rendering, to allow the measurement of light diffraction and prevent false positivity and background noise signals [30,31,32].

### 3.2. RNA In Situ Hybridization at the Cellular Level

As its name suggests, RNA in situ hybridization is the study of the fluorescent labelled RNA segment. This methodology is mainly used in the study of gene transcription at the cellular level [34,35]. In situ RNA-fluorescence hybridization (RISH) is a powerful tool for visualizing target RNA transcripts in cultured cells and tissue sections [36].

The main role of ribonucleic acid contains the encoding and decoding of genetic information, the ability to form different types of RNAs participating in gene expression, and gene regulation [37,38]. Many methods used for the visualization and imaging of each RNA type in cells and tissues are based on the understanding of cell-to-cell communication and on the understanding of the limitations and principles of synthesis and critical functions of RNAs in organisms, such as 3′-end polyadenylation in RNA stability and cancer development [37,39].

Researchers are currently focused on the detection of non-coding RNA (ncRNA), both long and short, to understand their role in organisms. Since the discovery of these small molecules in 1993, scientists have made remarkable progress in the identification and classification of small ncRNAs [40,41,42]. The common feature of these small nucleic acids is their length, which varies in range from 19 to 23 nucleotides in micro-RNA (miRNA), and from 26 to 33 nucleotides in PIWI-interacting RNA (piRNA) [34,43,44,45]. ncRNAs have a regulatory role at the post-transcriptional level which affects cellular processes at higher levels, and their deregulation can lead to undesirable processes with an impact on the whole organism. The consequence of such deregulations is the emergence and development of various diseases. Elucidation of the role of specific non-coding RNAs in the context of disease leads to the potential use of miRNAs as biomarkers [34,46].

#### 3.2.1. In Situ Hybridization Chain Reaction

**Figure 4:**
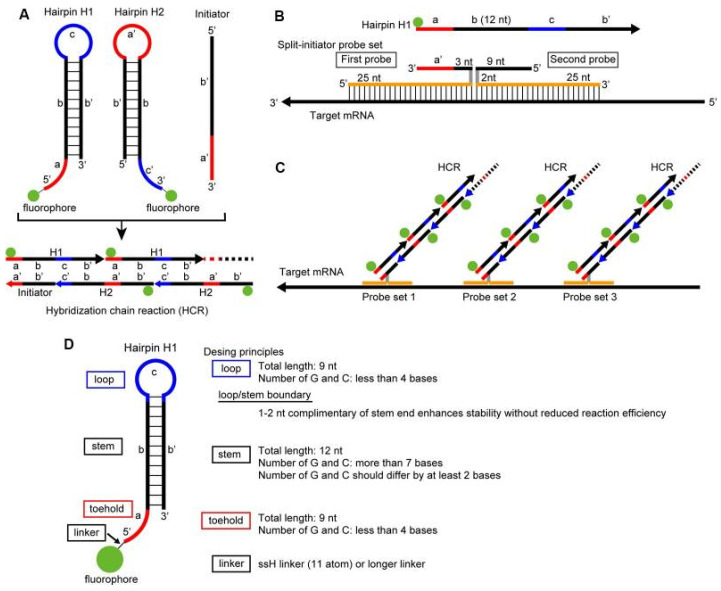
The principles of in situ hybridization chain reaction. (**A**) visualization of hybridization chain reaction (HCR) where metastable fluorescent harpins assemble into amplification polymers for the detection of initiators; the presence of an initiator triggers hybridization chain reaction. H1 complex nucleates with hairpin H2, providing the basis for a chain reaction alternating polymerization steps. (**B**,**C**) Every HCR protocol consists of two stages: detection and amplification stage. In the detection stage probes are hybridized to the target mRNA sequences and nonhybridized material is washed from the sample. In the amplification stage specially banded probes trigger fluorescent amplification polymers and every material present in the reaction which does not bind is washed; these washing steps minimalize the presence of background signals and nonspecific signals. (**D**) On C is visualized an experimental timeline of the reaction with the exact length of time of each step [47].

Various sensitive methods combining ISH and sequencing with painting methods have been developed for the imaging and detection of small RNA molecules [47]. In situ hybridization chain reaction (HCR) is revolutionary method combining the best characteristics of classic PCR with advance of in situ hybridization. We distinguish two main types of HCR—linear and nonlinear—divided into several subtypes—branched, dendritic, hydrogel-based clamped HCR, and others. HCR combines multiplexing, quantitation, sensitivity, resolution, and versatility. This isothermal enzyme-free nucleotide polymerization method is suitable for the detection of both RNA and DNA [48]. HCR is commonly used to detect mRNA in all kinds of cells and tissues, or in the whole organism. The third generation of the HCR methodology is establishing which method uses the same principles as other in situ hybridization techniques, but improves the probes and amplifiers involved in the reaction to provide the suppression of background by ensuring that each of the reagents, even if they bind non-specifically, will not amplify the background [48,49]. Reactions use two types of hairpin nucleotides, H1 and H2, which consist of the toehold, stem, and loop domains. A visualization of the in situ chain reaction and reaction protocol is in Figure 4 [49]. Domains are metastable and self-assembling when an initiator is not presented. With the initiator, domains hybridize to hairpin 1 through strand displacement and the remaining single strands hybridize with hairpin 2, producing a sequence identical to the initiator and the reaction starts again by forming a long nicked double helix [32,47]. A common problem observed during HCR reactions is the presence of false positive, non-specific, and background signals [47,48]. A partial solution for these issues is the use of split probes with a high signal-to-noise ratio, to increase the probe sensitivity, and fluorescently labelled RNA hairpins for the detection of a higher number of mRNAs [32,47]. Despite all the technical advantages of this methodology, it is not implemented in laboratories due to high financial costs [32,47,48]. However, in a recent study, Tsuneoka and Funato improve the HCR reaction by redesigning the harpins, shortening the length of the harpins and initiators using split probes, and removing the treatment with proteinase K. These approaches help to simplify the protocol and decrease the cost by approximately 66%, making this revolutionary method even more applicable for laboratory use in the visualization of multiple gene expressions. [47]. 

#### 3.2.2. miRNA In Situ Hybridization

Different forms of ISH are widely used in different fields of scientific research. Many techniques are used in the detection of expressions of single or more specific miRNAs such as northern blotting, qPCR, microarray, etc. [49]. The disadvantage of these methods is their inability to detect specific miRNAs at the cellular level [50]. For cellular detection, miRNA in situ hybridization (miRNA ISH) is used, which is almost identical to classical FISH, containing the same processing steps. Through the years, miRNA ISH has been improved by several modifications such as the use of locked nucleic acid (LNA) based probes, which reduce background signal and improve the signal of the probe [51]. The application of a double-labelled probe signal reduces the background noise ratio level (multicolor microRNA ISH). Carrying out an ethyl-3-dimethylaminopropyl carbodiimide hydrochloride (EDC-1) based fixation prevents free miRNA from escaping into the hybridization buffer [50,51] and the use of EDC-1 is causing the destruction of the epitope of the cell surface markers [49,51]. 

**Figure 5:**
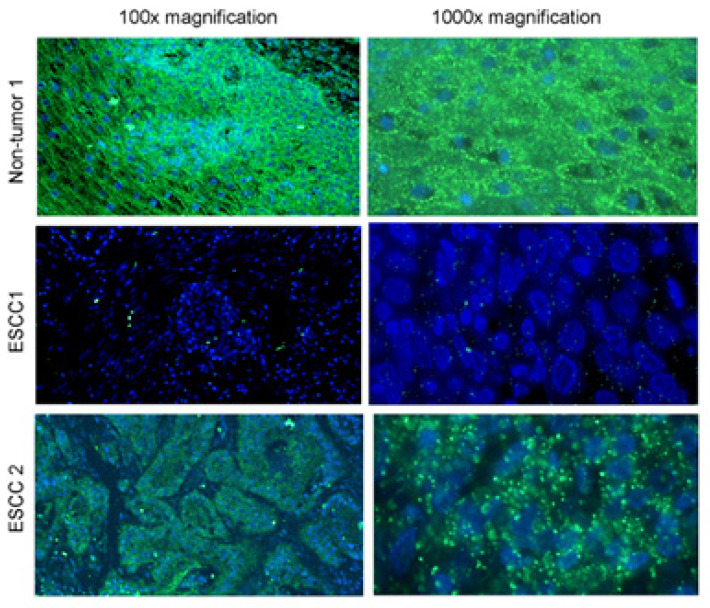
Multicolor miRNA ISH on non-tumor and cancer cells. In the picture is a visualized application of multicolor miRNA ISH for the detection of downregulation of a specific type of miRNA (miR-375) in Esophageal Squamous Cell Carcinoma and normal tissue on two level of magnification on left 100× magnification and on right 1000× magnification [53].

These features help to create a multicolor miRNA ISH protocol for the detection of tumor-specific miRNAs in samples. To avoid tissue permeabilization and miRNA loss, a longer hapten-probe linker was used; to prevent mishybridization, a judicious design of LNA-modified DNA probe was established. Use of directly labelled fluorescent rRNA ensured easy assessment of RNA retention, signal normalization, and simultaneous fluorescent detection of two miRNAs and rRNAs with increased multiplexing capacity [50,52]. Combining RNA sequencing and ISH technology is considered to be a powerful platform for identification and visualization of disease specific markers [49,50,52]. Specific use of miRNA ISH is in Figure 5, visualizing the expression of miR-375 in three types of tissue samples. The figure shows reduced expression of miR375 in tissue where Esophageal Squamous Cell Carcinoma (ESCC) was detected; this downregulation is linked with lymph node metastasis and poor overall survival rate in this type of carcinoma. In recent years many types of miRNA have played a role in regulation mechanisms in different kinds of oncological processes, and cancer progression was detected. The study of miRNA expression and regulation in tumor tissue may lead to the identification of miRNA serving as biomarkers to determinate the progression of a disease, and to rate the patient into the risk group due to the expression of specific miRNAs [53]. 

#### 3.2.3. Click Amplifying Fluorescent In Situ Hybridization

Another ISH method characterized by high specificity and intensity gain of signal amplification is Click Amplifying FISH (Clamp FISH). Currently, several types of in situ hybridization techniques focused on amplification are known, but each one of them suffers from a particular limitation [54]. In tyramide signal amplification, horseradish reacts with hydrogen peroxidase to cause a reaction, resulting in fluorochrome labelled tyramides accumulating at the side of hybrid [55,56]. It is frequently combined with another type of diagnostic procedure, such as enzyme-labelled fluorescent phosphatase substrate methods, or combined with immunofluorescence [54,55]. Enzyme labelled fluorescence forms a persistent product and is widely used in histochemical diagnostics as enzyme-mediated detection allows high resolution due to catalytic turnover of fluorogenic, chemiluminescent, or chromogenic substrates [56]. This level of sensitivity is needed for the histological samples’ significant backgrounds presented from either natural autofluorescence or fluorescence, created during preparation [55,56]. Both methods serve for the detection of proteins, viruses, and mRNA and their transcripts [57]. Another advantage of the Clamp FISH technique is its use in RNA detection by the RNA-based flow cytometry method [54].

**Figure 6:**
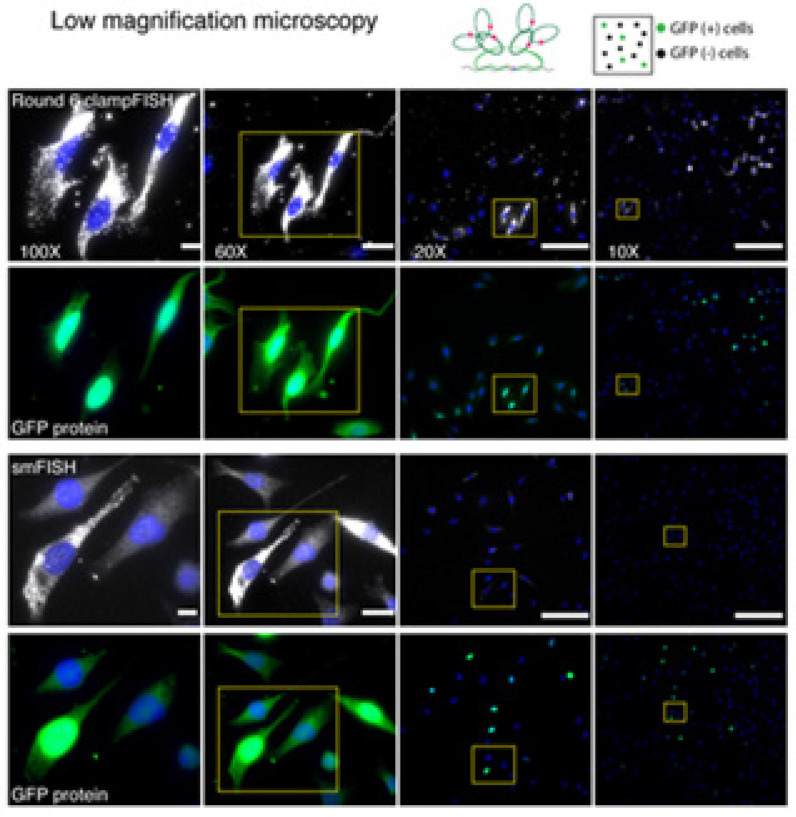
Application of Clamp FISH on population of cells. In the picture is shown the application of Clamp FISH in detection of GFP mRNA in diverse population of cells with expression or non-expression of examine gene. Cells are captured on different magnification levels [54].

In traditional single molecule RNA FISH (smRNA FISH), tissue samples suffer from high background levels contributed by a low signal-to-noise ratio. In smRNA FISH diagnostics, a high magnification microscopy to discern a positive signal from the background is crucial. This limitation led to the invention of new methodologies such as Clamp FISH (Figure 6), a type of non-enzymatic amplification reaction using enzymatic ligated padlock probes, a class of circular DNA probes, with high specificity and sensitivity to avoid non-specific binding and prevent spurious amplification [54,56,58]. The end of the strand is then connected with ligase, which wraps the molecule around the target strand following a click chemistry strategy to covalently link the ends of probes together, forming a loop around the target nucleic acid [55,58]. For exponential amplification, the backbone of the primary probe consists of two landing pads for a set of secondary fluorescence probes. On the backbone of the secondary probe, tertiary probe binding in a ratio of 2:1 hybridizes and doubles the signal in every round [54,56]. Using these principles and probe designs with other types of in situ hybridization allows for changes in the backbone sequence and the use of multiple amplifiers to study and detect multiple RNA targets [56,58].

#### 3.2.4. Click-Encoded Rolling Fluorescent In Situ Hybridization 

**Figure 7:**
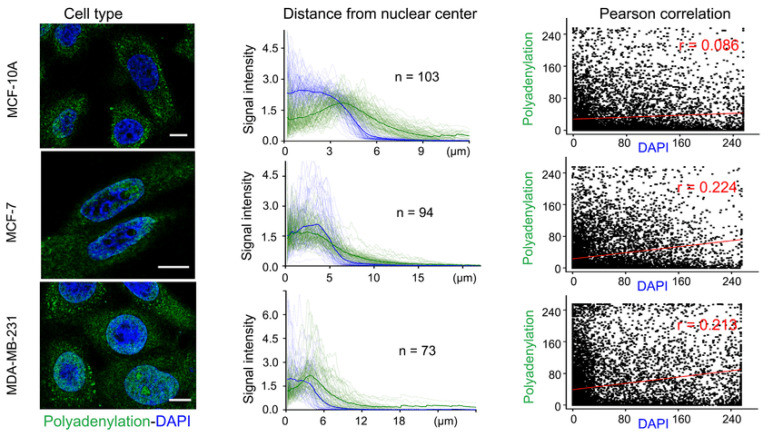
Specific organization of RNA polyadenylation in specific cells. The pictures in the left column visualize three types of cells, in the cells is polyadenylation identified with green-fluorescent dye and DAPI with blue. The middle column shows signal intensity of DAPI and adenylation as a function for the distance of polyadenylation from the nuclear center, and in the right column is this distance interpreted by Person correlation [59].

The Click-encoded rolling FISH (Clicker FISH) is a method based on the principle of translating cellular RNA features into chemical barcodes and spatially resolved images [59], labelling RNA poly-A tails and single strand and duplex regions with chemical tags in living and fixed cells [59,60]. The labelling of subcellular distribution is visualized by enzymatic amplification assisted FISH [59,61]. The Clicker FISH method applies padlock probes prepared by a multistep process containing hybridization, ligation, digestion, and three sets of clickable barcode primers to minimize non-specific binding [39,60,61]. Click-encoded rolling FISH uses three types of click chemistry reactions for encoding RNA features into fluorescence signals [59]. The enzymatic process of amplification is based on rolling circle replication of short DNA circles, allows us to visualize how poly-A tails protein exports from nuclei into cytoplasm, and displays a subcellular distribution of RNA [50,60]. This reaction can be modified to detect not only poly-A tails, but also for the detection of RNA modifications such as N6-methyladenosine and pseudo uridine [59,61]. After hybridization, amplicon is visualized like a bright spot (Figure 7), the intensity of which characterizes the number of labelled RNA molecules [39,59]. For the analysis of investigated RNA structure regions, it is necessary to integrate Clicker FISH with single-cell transcriptomics imaging methods based on sequential hybridization [59,60]. Figure 7 shows the specific organization of RNA polyadenylation in cells and the distribution in specific types of cell. This information can help us understand how trana script is organized during a cell cycle, and especially the intensity of the signal by identifying the decrease and increase in this signal during each phase of the cycle.

## 4. Quantitative Fluorescent In Situ Hybridization

**Figure 8:**
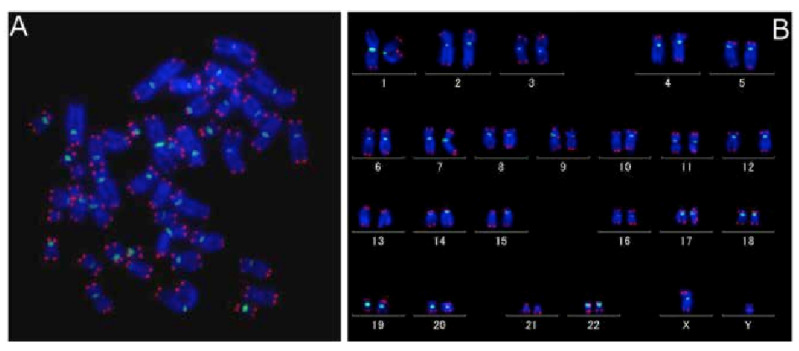
Quantitative FISH on metaphase spread (**A**) with human karyotype (**B**). Figure shows fluorescently dyed telomere (red) and centromere (green) on metaphase spread and then stored into the karyotype [62].

Quantitative FISH finds use in many fields of scientific research and in laboratory diagnostics. For the determination of the telomere length, Quantitative FISH is applied. The distal part of the chromosome is complementary to a fluorescent peptide-nucleotide probe-PNA, which can identify the length of the telomeres in the different types of cells in the examined tissues (Figure 8) [62]. In Figure 8, 46 human chromosomes are visualized with fluorescently labelled centromeres and telomeres to identify the process of shortening the telomere. For the detection of telomere length, another type of FISH is used: Primed in situ labelling (PRINS). Both quantitative FISH and PRINS methods have an advantage over classical Southern blotting, as blotting techniques are incapable of measuring length in the heterogeneous cell populations [24]. FISH helps in the identification of chromosomal instability in genomes by detecting aberrations such as ring chromosomes caused by many exogenous and endogenous factors. Telomeres are complexes of nucleoproteins playing an important role in protecting the terminal parts of the chromosomes, preventing their mutual fusion and DNA degradation [62,63]. The shortening of telomeres may be a consequence of multiple events [64]. These factors destabilize the chromosome by stretching its arms and causing breaks in the late phase of anaphase or telophase, therefore the shortening of telomeres is associated with early stages of oncogenesis, diseases associated with aging, diabetes mellitus, dementia, and atherosclerosis [65,66]. 

**Figure 9:**
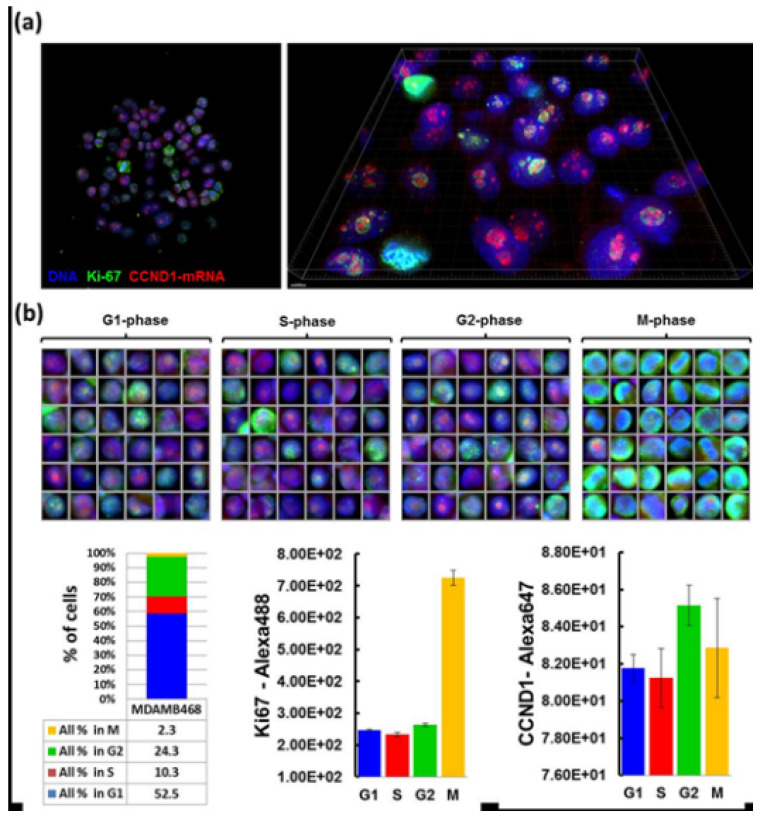
Application of Quantitative RNA ISH on breast cancer cells. (**a**) shows the nuclei of breast cancer cells visualized with two fluorescent dyes—green with gene Ki-67 and red with gene CCND1. The nuclei are seen on the left at 20× magnification, and on the right at high resolution. (**b**) shows numerous pictures of nuclei with monitored genes in each phase of the cell cycle. The panel in the table at the bottom of the picture contains quantitative analysis of gene expression, showing that the majority of expressions in gene Ki-67 are observed in mitotic phase, while in gene CCND1 there are the highest number of expressions in the G2 phase of the cell cycle [67].

Quantitative FISH is recently applied in the study of heterogeneity in transcription and the expression of genes and proteins in normal and tumor tissue. Due to the ability of this method to simultaneously quantify protein and the transcription level in cells of different origins, quantitative FISH is the right choice for determination of cell behavior in normal and affected tissue. Various types of quantitative ISH exist for the diagnostics of expressions of different kinds of genes able to control the cell cycle, or genes that are known for their significant role in oncogenesis and tumor suppression, illustrated in Figure 9. This shows interphase nuclei on two levels of magnification to demonstrate the amount of gene expression of Ki-67 and CCND1 in breast cancer cells. In recent years, quantitative RNA in situ hybridization has largely expanded due to its ability to detect and determine the amount of gene expression in special types of cells and tissues of interest [9,67]. 

## 5. Conclusions

Currently ISH is used in various fields of research and in different types of clinical applications; it is useful in many aspects of diagnostics for its ability to determine prognosis and relapse refractory. ISH methods can determine critical chromosomal translocation, deletion, and gains of genes or the whole chromosome that cause genetic diseases. This diagnostic tool also helps in the determination of suitable embryos in preimplantation diagnostics. Therefore, ISH is widely used in the field of prenatal and postnatal genetic diagnostics in humans. Based on the result of ISH diagnostics, and the presence of individual chromosomal aberrations, patients are stratified into a risk groups or, due to genetic findings, doctors can choose a proper therapeutic procedure and design a chemotherapy treatment. Specific use of this diagnostic tool is in preimplantation genetics to identify the aberration in embryos. ISH is helpful in the study of cell cycles, cell to cell communication, in the monitoring of gene expression, and in many other fields of scientific interest [68].

## Figures and Tables

**Table 1:** Classification of probe depending on labelling distribution.

Type of In Situ Technique	Application	Advantage	Probe
Interphase fish	Use in routine oncology diagnostic, haemato-oncology	Application on interphase cells—native or fixed	Locus specific DNA Probe
M-FISH SKY FISH COBRA FISH	Identification of unknown genetic material-marker chromosome—cancer diagnostic	Detection of complex changes in genome	Oligonucleotide chromosome-painting probe
In situ Hybridization Chain Reaction	Detect mRNA in all kinds of cells and tissues, or whole organism. Use in visualization of multiple gene expression	Combines multiplexing, quantitation, sensitivity, resolution, and versatility	Split-initiator DNA probe
Click Amplifying FISH-Clamp FISH	Detection of low abundance transcripts in tissue. Clamp FISH enables is flow cytometry-based measurement of RNA expression	Use multiple amplifiers to study and detect multiple RNA targets	Padlock probes
Click Encoded Rolling FISH	Understand how transcript is organized during cell cycle identifying decrease and increase in this signal during each phase of cycle	Displays a subcellular distribution of RNA	The circularized padlock probes
Mi-RNA ISH	Detect mRNA in all kinds of cells and tissues, or whole organism	Identification and visualization of disease specific markers	Locked nucleic acid (LNA) based probe
PRINS	Detection of long repeating sequences of DNA-satellites and telomeres	Capable of measuring the length in heterogeneous cell populations	Unlabeled, short and specific oligonucleotides
Quantitative FISH	Study of heterogeneity in transcription and expression of genes	Determination of cell behavior in normal and affected tissue	Fluorescent peptide- nucleotide probe-PNA-in telomere detection

## Data Availability

Not applicable.
